# Transarterial management of advance lung cancer

**DOI:** 10.1093/jjco/hyab050

**Published:** 2021-04-14

**Authors:** Shinichi Hori, Tatsuya Nakamura, Norifumi Kennoki, Ikuo Dejima, Atsushi Hori

**Affiliations:** Department of Radiology, Institute for Image Guided Therapy, Rinku Ohrai-mimami, Izumisano, Osaka, Japan; Department of Radiology, Institute for Image Guided Therapy, Rinku Ohrai-mimami, Izumisano, Osaka, Japan; Department of Radiology, Institute for Image Guided Therapy, Rinku Ohrai-mimami, Izumisano, Osaka, Japan; Department of Radiology, Institute for Image Guided Therapy, Rinku Ohrai-mimami, Izumisano, Osaka, Japan; Department of Radiology, Institute for Image Guided Therapy, Rinku Ohrai-mimami, Izumisano, Osaka, Japan

**Keywords:** lung neoplasms, embolization, therapeutic, anti-neoplastic agents, bronchial arteries

## Abstract

Previous reports on transarterial treatment for lung cancer were reviewed. The bronchial arterial infusion therapy has a long history since 1964. Better local control with less doses of anti-neoplastic agents was warranted by trying transarterial administration to lung and mediastinal tumors. It is reported that both primary and metastatic tumors are fed by bronchial or other systemic arteries. The bronchial arterial embolization for hemoptysis has been introduced for clinical practice since 1973. Hemoptysis by not only benign but also malignant diseases has been well controlled by embolization. In recent decades, the technical elements for transarterial treatments have markedly improved. They make it possible to carry out precise procedures of selective catheter insertion to the tumor relating arteries. Current concepts of transarterial treatment, technical aspects and treatment outcomes are summarized. Tentative result from chemo-embolization for advanced lung cancer using recent catheter techniques was also described. It provides favorable local control and survival merits. It is considered that a population of lung cancer patients can benefit from transarterial management using small doses of anti-neoplastic agents, with less complications and less medical costs.

## Teaser

Previous trials of transarterial treatment of lung cancer were reviewed and the current status of arterial chemo-embolization for advanced lung and mediastinal tumors was investigated.

## Background

On the basis of statistics by National Cancer Center Japan, lung cancer is the most common neoplasm of the highest incidence with the highest mortality. In total, 120 people out of 10 000 people are died of lung cancer in 2019. Furthermore, the incidence is getting higher with aging (1).

Locoregional therapies including endoscopic surgery or stereotactic radiotherapy are effective in very early-stage diagnosed by precise computed tomography (CT) examination, but in general the mortality of lung cancer in rather advanced stages is the highest among all cancers. Especially concomitant diseases in aged patients disturb extensive therapies recommended by the treatment guideline. Generally, patients with recurrent diseases after standard therapies are in advance stages. Introduction of additional systemic chemotherapy does not contribute to advanced or aged patients (2). The treatment of lung cancer in these states is always palliative in keeping better performance status.

Recent advancement of molecular target agents sometimes presents drastic effects. However, unexpected side effects are the new problems to be solved. Application of new drugs including immune checkpoint agents requires tremendously high cost. It may raise financial problems to medical resources. Radiotherapies supported by recent technology including photon or heavy ion therapy have similar problems.

In this clinical situation, more effective treatment for advanced lung cancer in addition to standard therapies is required. For patients in these stages, it is important to select a treatment method that offers the optimal control of tumor growth, limited adverse events, lower medical cost and minimal disruption of their life. Transarterial chemo-embolization therapy for primary advanced lung cancer seems to fulfil these criteria and can be regarded as an alternative to intravenous cytotoxic treatment with minimum disturbance of the quality of life. Unfortunately, this method is not well-known as the clinical practice. However, clinical trials to administrate anti-neoplastic agents to pulmonary tumors through systemic arteries have already started since 1964 (3). The aim of the trials was to avoid systemic toxicity, increase in the dose and concentration of antitumor agents in target lesions (4–7). Selection of proper drugs for individual tumor histopathology is one of the current problems to be solved by clinical evaluation. Doses of drugs can be decided according to the target tumor volume. Embolization of bronchial artery (16) was introduced in 1973 as a treatment of hemoptysis caused by benign diseases. It has also been introduced for neoplastic lesions to control hemoptysis. It is now recognized as the first treatment option for life threatening hemoptysis.

One of the reasons that this treatment has not been recognized as a standard method was the technical difficulties. Recently the technical elements of transarterial treatments have been improved thanks to medical technologies of late years. In this paper, the history of transarterial management of lung cancer was reviewed and present status of endovascular approach for advanced lung cancer was investigated. Finally, the clinical trials and tentative results for advanced lung cancer in our institute will be presented.

## History of trasnarterial infusion

The first systematic research work for the bronchial arteriography done by femoral arterial approach was reported in 1964 by Viamonte (3). Basic anatomies and angiographic findings were investigated**,** suggesting the possibility of trans-bronchial arterial treatment for malignant tumors (3). In 1965, Kahn et al. (8) did a clinical trial in 25 patients and reported usefulness of bronchial infusion of methotrexate for neoplasms in getting temporary palliation, on the concept that pulmonary neoplasms and lung tissues receive their arterial blood supply from the bronchial arteries (9,10). In 1969, Neyazaki et al. (4) reported on bronchial infusion therapy in 27 patients chiefly as surgical adjuvant chemotherapy using Mitomycin C and 5-FU. They described that marked shrinkage of lung cancer on x-ray films was found in half of the patients without serious side effects. Many reports recommended repeated treatments to get better results (4,6,7).

In spite of favorable and promising results of early clinical trials, transarterial treatment of lung cancer did not become a standard therapy. There may be many reasons. Treatment technic was usually complicative and success rate was affected by many factors including physician’s skill, capabilities of angiography machine and catheter devices (11). During these several decades, many of anti-neoplastic agents, molecular target agents and immune check point inhibitors have been introduced into clinical practice. New technologies for radiotherapy promised better local control. However, there still are many patients with advanced lung cancer who cannot have benefits from new medical technologies for various reasons.

Nevertheless, several trials of transarterial treatments (5,6,10–15) have been continued in order to get better treatment results for advance lung cancer. Many reports described better local effects with low toxicity (4,8–11,15) due to low doses compared with systemic doses. These trials convey the importance of transarterial chemotherapy and the necessity of reappraising the transarterial management of advanced lung cancer using recent technologies for transarterial treatments (7).

## History of embolization therapy

The first trial of bronchial artery embolization for hemoptysis was reported in 1973 by Remy et al. (16). It has been established as the first option of treatment for benign life threatening hemoptysis caused by benign diseases including bronchiectasis, cystic fibrosis or tuberculosis (17). During clinical practice, hemoptysis in lung cancer has been included in the indications. It was estimated that ~30% of lung cancer patients develop hemoptysis during their clinical course (18) and out of these, 10% will experience massive hemoptysis (19). Due to the decrease of tuberculosis cases, lung cancer has become the most frequent cause of pulmonary hemorrhage in recent years (20). Hemoptysis caused by neoplasms is considered good indication to start bronchial embolization (17,19–23). The clinical success rate, which means cessation of hemoptysis, was higher than 80% (17,20,23). Nevertheless, mortality resulting from hemoptysis and recurrence rate is high among these patients secondary to progression of lung cancer (21,24). On the other hand, prolongation of survival time by bronchial embolization was attributed to cessation of hemoptysis. A consistent embolization strategy is important to get good results (20). The control of growth of lung cancer seems very important to get better results.

In recent years, combination of embolization and arterial infusion were reported for cases with metastatic tumors (25–27). Recent technology of embolization allows us to use drug-eluting microspheres to get tumor regression (28).

## Present understanding on arterial management of lung cancer

In the long history of transarterial approach to lung cancer, many of knowledges and experiences have been accumulated.

### Catheter techniques

Sometimes catheter insertion to bilateral subclavian arteries is necessary. For this reason, transfemoral approach is preferable to transradial approach for lung cancer treatment. A coaxial catheter system composed of 4 Fr guiding catheter and 2 Fr microcatheter is recommended in selecting small multiple branches feeding lung tumors (21). Navigation using microguidewire is not recommended in preventing vascular spasm that makes it difficult to get better drug distribution. In caour facility, we use a pre-shaped microcatheter to select bronchial arteries or small branches arising from subclavian artery without vascular spasm. Instead of aortography, precise analysis of dynamic CT images is useful in identifying individual anatomical variations (17,29). Vascular 3-D images effectively support selecting small branches arising from the aorta (30). Selective arterial contrast infusion CT is crucial in estimating individual arterial supply to the target lesions (11,26,27).

### Circulation of lung tumors

Birnbaum (9) stated that the lung tumors are fed by the bronchial artery. This testament is the fundamental issue for transarterial treatment of lung and mediastinal tumors (3,4,8). It is well supported by the recent medical technology when doing CT scan during individual arterial contrast infusion. Almost all primary and metastatic tumors in the lung are fully enhanced from systemic arteries (25–27). The tumors in the lung field are mainly supplied by bronchial arteries. When they invade mediastinum, the internal thoracic, inferior phrenic and branches from subclavian artery may feed the tumors through various anastomosis (6,11). The tumors invading chest wall are fed from intercostal arteries or chest wall branches from subclavian artery (11). All feeding arteries should be thoroughly examined to detect tumor staining for better treatment effects (11). There are lot**s** of anatomical variations in bronchial arteries (3,8,10). Patients after surgery or radiotherapy may have various reconstructed arteries caused by vascular damages.

### Angiographic and CT findings

Tumors in the lung and mediastinum are generally visualized as tumor stain (3,8,11,17,23) in angiography. Tumor stain is the most important factor in obtaining a successful outcome (11).

Dilatation of tumor feeding arteries is usually found (6,8,17,23). However, the incidence of vascular abnormality is lower than in benign diseases. Shunting into pulmonary artery or vein is commonly found (6). Embolization therapy should be avoided in the case of early filling to pulmonary vein in angiography (17,24). Arterial infusion CT is much reliable in evaluating the vascular supply to the tumors. It is very common to find good enhancement on CT even if angiography did not show enhancement of tumors (24).

### Anti-neoplastic agents

Many kinds of drugs including Methotrexate (8), Mitomycin C (4–6), 5-FU (4), Vincristine (5), Beomycin (5), CDDP (2,11,13), Gemcitabine (11) and Doxorubicin (11) have been used since 1965. Doses of other chemotherapeutic agents were usually limited to less than half of those used in systemic chemotherapy (11). Recently, CDDP has been the most common drug for arterial infusion. Further suitable drugs might be applied in the future. In cases of multiple feeders, the total dose was divided according to the degree of tumor staining in each artery (11). New drugs including molecular target drugs and immuno-checkpoint inhibitors have not been reported in previous reports.

### Embolic agents

Embolizations have been performed using a variety of agents. Polyvinyl alcohol particle and gelatin sponge (17,22) are the most common materials at present. However, they cause vascular damages and make it difficult to repeat treatments. Metallic occlusion coils are not recommended because they might cause proximal occlusion that makes it difficult to repeat the same procedure. Liquid materials including *N*-butyl cyanoacrylate or Lipiodol may be unsuitable, for they cause damage to bronchial wall or esophagus. Many recent reports (24–26,28) recommend calibrated microspheres which provide effective and safe embolization of peripheral arteries. In cases of lung and mediastinal tumors, ischemic effect is not enough to evoke necrosis by microspheres. However, the role of embolic material is to help retention of anti-neoplastic agent within the target lesions. For this purpose, microsphere is beneficial for safer and effective embolization therapy. Recently many kinds of drugs can be loaded on polyvinyl-alcohol or superabsorbent polymer**-**based microspheres. Long**-**term continuous release of drugs within the tumor can be expected.

### Complications

Chest pain and pyrexia are the most common minor complications of bronchial arterial embolization, with a reported prevalence of 20–50% (4,11,17,20,21,23). Both symptoms are likely related to an ischemic phenomenon caused by embolization and are usually transient. No serious adverse events that cause respiratory function damages were reported (5–7,11). Esophageal ulcer was reported with MMC infusion when combined with radiotherapy (6). Bronchial ulcer caused by high dose of cisplatin was reported (13). Although the maximum tolerated and appropriate dose are not clearly known (13), 50 mg of cisplatin may be the highest dose to prevent local side effects (11). Doses of the other chemotherapeutic agents were limited to less than half of those used in systemic chemotherapy to avoid systemic side effects (11). The most serious complication reported was spinal cord injury (23). Recent advanced anigo-CT is definitively important to recognize circulation to the spinal cord. The second problem of embolization is direct shunting from bronchial artery into pulmonary artery or pulmonary vein (6,24). It is difficult to differentiate pulmonary venous shunting from pulmonary artery shunting. Particle embolic material can go into systemic arteries through bronchial artery to pulmonary vein shunting. Careful observation on angiography images is necessary. During the procedure of embolization, careful attention is necessary by monitoring overflow of embolic material from the targeted artery. Tight collusion by embolic material should be avoided to prevent permanent occlusion of bronchial arteries.

### Local control and prognosis

Many previous reports (4–7,11) indicate the apparent tumor regression (20–50% in size) after trans-arterial chemo-infusion. However, anti-neoplastic agents used were different from those used in current regimen. Although It seems difficult to compare the results with recent data, regression of lung and mediastinal tumors can sufficiently be expected. Repetition of treatment improved the treatment results (4,6,7). Bronchial arterial infusion as a neoadjuvant therapy was feasible and effective with no treatment-related complications (7). There was no comparative data as to the prognosis, but the tumor regression may contribute to improve prognosis of advanced lung cancer (2,7,11,12,15).

There were no reports proving tumor reduction of lung cancer by embolization therapy. A recent study using drug-eluting beads reported better local response even in small number (28).

Repeated treatment was recommended because improvement of prognosis was attributed to control of hemoptysis (20,24).

**Figure 1.**
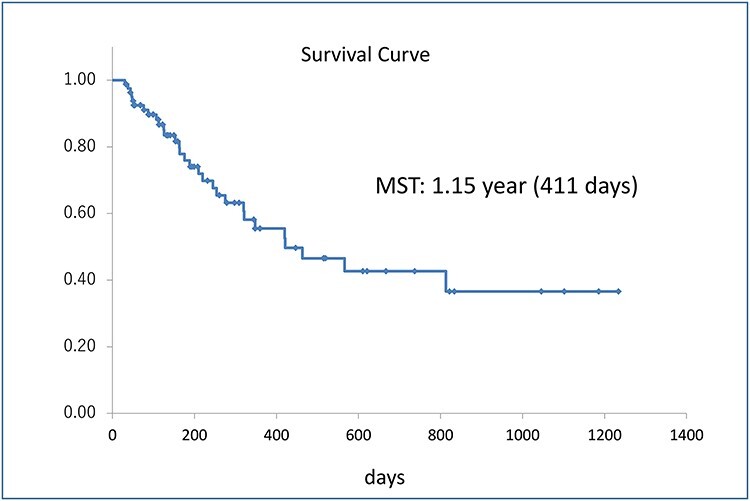
Cumulative survival curve of 82 patients with advanced lung cancer. Median survival time was 1.15 months.

**Figure 2.**
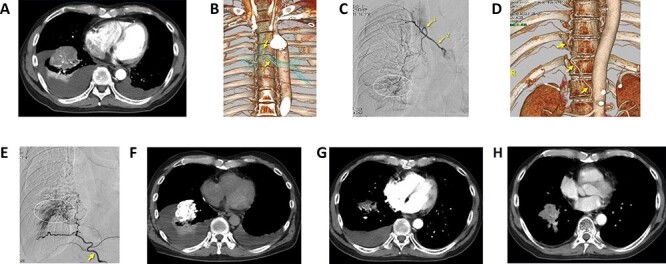
(A) Dynamic computed tomography (CT) in arterial phase. The tumor in the right lower lobe was adjacent to the diaphragm. Partial atelectasis of right lower lobe and pleural effusion were found. (B) A 3-D image was reconstructed from dynamic CT. The right bronchial artery (arrow 1) arises from the common trunk with intercostal artery (arrow 2). (C) Arteriography of right common trunk (arrow 2) and bronchial artery (arrow 1). A vague tumor stain (circle) was found in the right lower lobe. (D) A 3-D image of the right inferior phrenic artery (arrows). (E) Arteriography of the right inferior phrenic artery (arrow). Tumor stain (circle) was clearly found. (F) CT during selective arterial infusion into the right inferior phrenic artery. The tumor was clearly enhanced through the diaphragm. A total of 20 mg of cisplatin, 20 mg of docetaxel, 250 mg of fluorouracil and 100 mg of bevacizumab were infused through the right bronchial artery and right inferior phrenic artery. After infusion, embolization using 2.0 mg of Adriamycin loaded HepaSphere was done. (G) CT in 3 months after the initial therapy. Three sessions of treatment had been done, marked tumor regression was confirmed. No serious complications were found during and after the treatment. Respiratory symptoms were improved. (H) CT in 1 year after the initial treatment nine sessions of treatment had been done. The tumor was stable in size. The right pleural effusion had disappeared.

## Technical aspects

Recent medical technologies provide excellent CT images including 3-D reconstruction. Endovascular treatments can be performed safely and effectively under the sophisticated angio-machines sometimes combined with CT scan. Selective catheter insertion is safely and successfully done using well-designed microcatheters. Thanks to these excellent products, trans-arterial management of pulmonary neoplasms has been gradually accepted with good results (24–26,28). However, there is no discussion about choosing the best drugs and administration methods for this treatment method. The best treatment method should have the least adverse events with the least medical costs in providing it. Moreover, the technology in the other medical field including locoregional immuno-oncology (31) should be combined to get better results for lung and mediastinal tumors.

## Clinical endpoints

Bronchial arterial infusion may help patients to control annoying symptoms. Improvement of respiratory symptoms was reported after bronchial infusion (4). At least, progression of respiratory symptoms after treatment has never been reported.

As to hemoptysis, it is well controlled (77.5–100%) immediately after bronchial arterial embolization (20,21,23) even in patients with lung cancer. It is strongly recommended to introduce embolization therapy as the first treatment option (20,21,23) for patients with hemoptysis.

**Figure 3.**
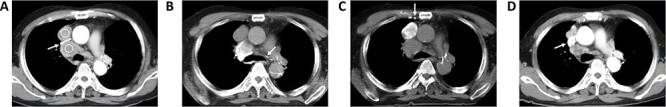
(A) Contrast enhanced CT before treatment. Stenosis of superior vena cava (SVC, arrow) was caused by lymph node metastases (circles). (B) CT during contrast infusion through the right bronchial artery (arrow). Lymph node metastasis posterior to SVC was enhanced. Cisplatin (20 mg) and 5-FU (200 mg) infusion followed by embolization with HepaSphere (100–150:1.5 mg) was performed. (C) CT after 2 sessions of treatment. Re-opening of SVC (arrow) due to shrinkage of lymph node metastases was found. The SVC syndrome was completely improved. (D) CT after two sessions of treatment. Re-opening of SVC (arrow) due to shrinkage of lymph node metastases was found. The SVC syndrome was completely improved.

**Figure 4.**
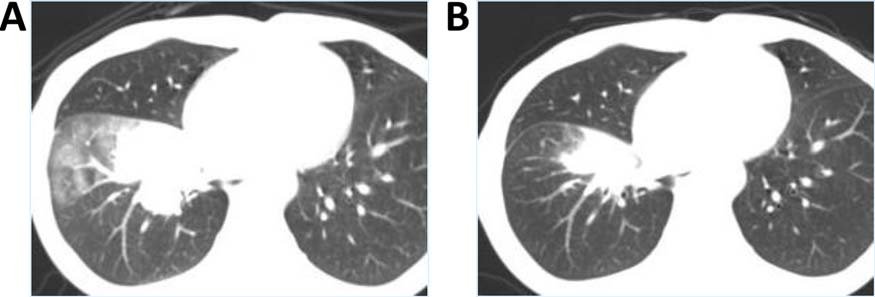
(A) CT before treatment. Obstructive pneumonia was found in the right lower lobe. Transarterial chemo-embolization through two right bronchial arteries and right inferior phrenic artery was performed using 30 mg of cisplatin and 500 mg of 5-FU with 5 mg of HepaSphere (50–100). (B) CT in 2 months after treatment. Obstructive pneumonia was markedly improved according to shrinkage of tumor invading the pulmonary hilum. Symptoms caused by obstructive pneumonia were improved.

From analysis of previous reports, the chemo-embolization of bronchial and relating arteries is feasible and beneficial for patients who are suffering from respiratory symptoms making it easier for them to continue effective treatments to obtain better prognosis.

## Results

A total of 82 patients with advanced lung cancer were treated by trans-arterial method between August 2016 and December 2019. Enrolled patients with advanced lung cancers were unresponsive or incompatible to standard chemotherapy and surgery. The purpose of treatment was improvement of symptoms and extension of life combining local chemo-infusion and arterial embolization therapy. Prior to treatment, 3-D arterial images from dynamic CT scan by bolus venous contrast injection were reconstructed. A 4.0 F guiding catheter was advanced through the femoral artery to the thoracic aorta under local anesthesia. A 2.0 microcatheter was advanced coaxially to the bronchial arteries and other tumor branches from subclavian artery or inferior phrenic artery. Selective arterial infusion CT was done to confirm the blood flow to the tumors. Anti-neoplastic agents were 5-FU, cisplatin, docetaxel, irinotecan, and/or bevacizumab. Each dose was 10–20% of systemic chemotherapy. The embolic material was one of spherical embolic materials; HepaSphere ranging 50–100 microns. Embolization was carried out immediately after infusion of anti-neoplastic agents to help retention of drugs in the target lesions. The embolization was terminated when reduction of arterial flow was observed under fluoroscopy. The procedure time was within 2 h.

There were no serious complications. Special attentions were paid to avoid embolization of the anterior spinal artery. Respiratory symptoms and chest pain were improved after treatments in more than half of the patients. Treatments were repeated on demand.

A survival curve of 82 patients with advanced lung cancer is in Fig. 1. Median survival time was 1.15 year (411 days).

## Discussion

It is hard to compare with other treatment results because there are no comparative data with advanced lung cancer. Median survival time, 1.15 months, is better than we expected. Less invasiveness of this treatment may be the main reason for prolonged prognosis. We currently assume that the trasnarterial management of advanced lung cancer has a significant role in controlling clinical symptoms, extending patients’ life term with less adverse events and less medical costs. However, the clinical results depend on the quality of treatment apparatus and physician’s skill. Training and research facilities for transarterial treatment should be established to make it prevalent in the clinical field of oncology. Limitation of this study was that this was a single center retrospective study. A prospective multicenter study is necessary to persuade clinical value of this treatment.

### Case presentation

**Case 1** (Fig. 2)

A 63-year-old male with right lung adenocarcinoma cT2bN2M1. After systemic chemotherapy using CBDCA, PEM, S-1, DOC and RAM, tumor progression was observed. Symptoms were dull pain in chest wall and severe cough.

**Case 2** (Fig. 3)

A 63-year-old male with mediastinal lymph node metastases of recurrent squamous cell carcinoma after surgery. Adjuvant chemotherapy using cisplatin and vinorelbine was interrupted because of leukocytopenia. After 1 year he had superior vena cava syndrome caused by mediastinal lymph node metastases.

**Case 3** (Fig. 4)

A 43-year-old female with recurrent adenocarcinoma in the right lower lobe. She had been treated with systemic chemotherapy for 3 years. She had high fever, cough and hemosputum caused by obstructive pneumonia.

## Conclusion

The transarterial treatment for lung cancer has been developed since 1964. Although it is not considered as the standard method for many reasons, recent medical technologies are making it a practical and effective treatment for advanced lung cancer. In the next decade, much more effective agents will be introduced in the field of oncology, but new kinds of adverse events and higher medical costs may be added as issues to be solved. It is considered that a population of lung cancer patients can benefit from transarterial management. Multicenter clinical study with a large cohort in collaboration with medical oncologists and interventional radiologists is necessary.

## Supplementary Material

References_final_version_hyab050
