# Supplementary material for: Transarterial management of advance lung cancer
Source: Jpn J Clin Oncol. 2021 Apr 14;51(6):851–6. doi: 10.1093/jjco/hyab050 (PMC8163058; doi:10.1093/jjco/hyab050)
Supplement: References_final_version_hyab050 [file references_final_version_hyab050.doc]

References

1 Cancer statistics in Japan-2019 <https://ganjoho.jp/data/reg_stat/statistics/brochure/2019/cancer_statistics_2019_fig_J.pdf>

2 Ieki R The treatment selection of elderly lung cancer patient. Jpn J Lung Cancer Clin 2000;3:147-155.

3 Viamonte Jr M. Selective bronchial arteriography in man (preliminary report). Radiology 1964;83:830-839.

4 Neyazaki T, Ikeda M, Seki Y, Egawa N, Suzuki C. Bronchial artery infusion therapy for lung cancer. Cancer 1969;24:912-922.

5 Hellekant C, Svanberg L. Bronchial artery infusion of mitomycin-C in advanced bronchogenic carcinoma. Acta Radiol 1978;17:449-462.

6 Ekholm S, Albrechtsson U, Tylen U. Bronchial artery infusion of mitomycin C in carcinoma of the lung. Cardiovasc Intervent Radiol 1983;6:86-96.

7 Osaki T, Oyama T, Takenoyama M, et al. Feasibility of induction chemotherapy using bronchial arterial infusion for locally advanced non-small cell lung cancer: a pilot study. Surg Today 2002;32:772-778.

8 Kahn PC, Paul RE, Rheinlander HF. Selective bronchial arteriography and intra-arterial chemotherapy in carcinoma of the lung. J Thorac Cardiovasc Surg 1965;50:640-647.

9 Birnbaum, G. L.: Anatomy of the Bronchovascular System; Its Application to Surgery, Chicago.The Year Book Publishers, Inc 1954;64:872.

10 Hellekant C, Jonsson K Double blood supply of bronchogenic carcinoma from multiple arteries. Acta Radiologica Diagnosis 1981;22:403-406.

11 Nakanishi M, Demura Y,Umeda Y, Mizuno S, Ameshima S, Chiba Y, Ishizaki T. Multi-arterial infusion chemotherapy for non-smal cell lung carcinoma – Significance of detecting tumor feeding arteries and tumor staining. Lung Cancer 2008;61:227-234.

12 Watanabe Y, Shimizu J, Murakami S. Reappraisal of bronchial arterial infusion therapy for advanced lung cancer. Jpn J Surg 1990;20:27–35.

13 Osaki T, Hanagiri T, Nakanishi R, et.al. Bronchial arterial infusion is an effective therapeutic modality for centrally located early-stage lung cancer. Result of a pilot study. Chest 1999;115:1424-1428.

14 Koshiishi H, Utsumi K, Tamamoto F, Takahashi E. Evaluation of bronchial arterial infusion (BAI) for high risk lung cancer. Jpn J Cancer Chemother 2000;27:1907-1910.

15 Nakanishi M, Umeda Y, Demura Y, Ameshima S, Chiba Y, Miyamori I, Ishizaki T.

Effective use of multi-arterial infusion chemotherapy for advance on-small cell lung cancer patients: four c specified cases Lung Cancer 2007;55:241-247.

16 Remy J, Voisin C, Ribet M, et al. Treatment, by embolization, of severe or repeated hemoptysis associated with systemic hypervascularization. Nouv Presse Med 1973; 2:2060 [in French]

17 Fujita T, Tanabe M, Moritani K, et al. Immediate and late outcomes of bronchial and systemic artery embolization for palliative treatment of patients with nonsmall-cell lung cancer having hemoptysis 2014;31:602-7.

18 Hirshberg B, Biran I, Glazer M, Kramer MR. Hemoptysis: etiology, evaluation, and outcome in a tertiary referral hospital. Chest 1997; 112:440 – 444

19 Park HS, Kim YI, Kim HY, Zo JI, Lee JH, Lee JS. Bronchial artery and systemic artery embolization in the management of primary lung cancer patients with hemoptysis. Cardiovasc Intervent Radiol 2007; 30:638–643.

20 Witt Ch, Schmidt B, Geisler A, et al. Value of bronchial artery embolisation with platinum coils in tumorous pulmonary bleeding 2000;36:1949-54.

21 Garcia-Olivé I, Sanz-Santos J, Centeno C, et al. Results of bronchial artery embolization for the treatment of hemoptysis caused by neoplasm. J Vasc Interv Radiol 2014;25:221-228.

22 Hayakawa K, Tanaka F, Torizuka T, et al. Bronchial artery embolization for hemoptysis: immediate and long-term results. Cardiovasc Intervent Radiol 1992;15:154-159.

23 Wang G, Ensor JE, Gupta S, et al. Bronchial artery embolization for the management of hemoptysis in oncology patients: utility and prognostic factors J Vasc Interv Radiol 2009;20:722-729.

24 Lorenz JM, Navuluri R. Embolization of chest neoplasms: The next frontier in interventional oncology. Semin Intervent Radiol 2019;36:176-182.

25 Seki A, Hori S, Sueyoshi S, et al. Transcatheter arterial embolization with spherical embolic agent for pulmonary metastases from renal cell carcinoma. Cardiovasc Intervent Radiol 2013; 36:1527–1535.

26 Kennoki N, Hori S, Yuki T, Hori A. Transcatheter arterial chemoembolization with spherical embolic agent in patients with pulmonary or mediastinal metastases from breast cancer. J Vasc Interv Radiol 2017; 28: 1386–94.

27 Hori [A,](https://www.birpublications.org/author/Hori%2C+Atsushi) [Ohira](https://www.birpublications.org/author/Ohira%2C+Ryosuke) R,  [Nakamura](https://www.birpublications.org/author/Nakamura%2C+Tomoyuki) T, et al. Transarterial chemoembolization for pulmonary or mediastinal metastases from hepatocellular carcinoma. [doi.org/10.1259/bjr.20190407](https://doi.org/10.1259/bjr.20190407) BJR 2020:93

28 Bie Z, Li Y, Li B, et.al. The efficacy of drug-eluting beads bronchial arterial chemoembolization loaded with gemcitabine for treatment of non-small cell lung cancer. Thoracic Caner 2019;10:1770-1778.

29 Mori H, OhnoY, Tsuge Y, et al. Use of multidetector row CT to evaluate the need for bronchial arterial embolization in hemoptysis patients. Respiration 2010;80:24-31.

30 Hayes Jr D, Winkler MA, Kirkby S. Capasso P, Mansour HM, Attili AK Preprocedural planning with prospectively triggered multidetector row CT angiography prior to bronchial artery embolization in cystic fibrosis patients with massive hemoptysis. Lung(2012) 190:221-225.

31 Lucatelli P, Iezzi R, De Rubeis G, et al. Immuno-oncology and interventional oncology: a winning combination. The latest scientific evidence. Eur Rev Med Pharmacol Sci 2019;23:5343-5350
